# Haste and Health

**Published:** 1889-07

**Authors:** S. H. Preston


### HASTE AND HEALTH.

BY S. H. PRESTON.

Now a-days, men begin to die before they learn how to live. There are no more Methuselahs. The race is being railroaded along at steam speed. People are too hurried to think of health—are under too much pressure to pause for physiology. They bolt their meals, race for the cars, jump for the boat.

Those who live fast do not live well. The steady, moderate, methodical man does more work and better than one who tries to do in a day the work of a week. The racer gives out sooner than the plodding draught-horse. There is nothing which can be won by work in this world that can make amends for shortened and enfeebled lives.

The farmer who hurries to his field by early dawn is a fool. The mechanic who works after dark and seven days in the week is a fool. They are simply wasting nature's reserve fund of force, wasting all that is worth living for in this world. They are not only fools, but sinners. Right-living is the only rewarded righteousness on earth. Disease is the devil of this world.

Now, there is no gain in haste and over-work in the long run. The farmer gets tired and gives out at noon. The mechanic is soon unable to sleeps nights, and fails at forty-five. The student who sits up nights ere long becomes disqualified for study. He shakes and walks totteringly. His constitution becomes shattered, and he is obliged to take to his bed. Fret and worry, disease and death, are unprofitable returns for an effort to force nature.

Yet there are people who take things too easy to even keep in good health. There is nothing that shortens life like laziness. A certain amount of nervous stimulus is needful for longevity. A person's life may be so peaceful and his cares so few as to vitiate his vitality. He may decay and become prematurely old from sheer lack of employing his energies, while an active, bustling, business man may be in the full plenitude of his strength.

The Pitcairn Islanders, descendents of the mutineers of the Bounty, are an illustration. Living in a land that supplies them all the luxuries of life spontaneously, needing to make no exertion except for enjoyment, free from all anxiety, they begin to age at thirty, and die of natural decay at forty. Among other men similarly situated, those who take part in the government, the architects, astronomers and priests, live almost twice as long as the common people. An extreme of rust is as injurious as one of rush.

